# Supplementary material for: Mitochondrial antiviral-signalling protein is a client of the BAG6 protein quality control complex
Source: J Cell Sci. 2022 May 11;135(9):jcs259596. doi: 10.1242/jcs.259596 (PMC9264363; doi:10.1242/jcs.259596)
Supplement: Reviewer comments [file joces-135-259596_review_history.pdf]

Mitochondrial antiviral-signalling protein is a client of the BAG6 protein quality control complex

Peristera Roboti, Craig Lawless and Stephen High
DOI: 10.1242/jcs.259596

Editor: David Stephens

Review timeline

Original submission:	2 December 2021
Editorial decision:	28 February 2022
First revision received:	29 March 2022
Accepted:	1 April 2022

Original submission

First decision letter

MS ID#: JOCES/2021/259596

MS TITLE: Mitochondrial antiviral-signalling protein is a client of the BAG6 protein quality control complex

AUTHORS: Peristera Roboti, Craig Lawless, and Stephen High
ARTICLE TYPE: Research Article

We have now reached a decision on the above manuscript.

To see the reviewers' reports and a copy of this decision letter, please go to: https://submit-jcs.biologists.org and click on the 'Manuscripts with Decisions' queue in the Author Area. (Corresponding author only has access to reviews.)

As you will see, the reviewers raise a number of criticisms that prevent me from accepting the paper at this stage. The majority of these can readily be addressed by changes to the text and, in some cases, the figures. I do however agree that the suggestion to examine mitochondrial and microsome fractions separately is a good one and would strengthen the manuscript, at least in terms of those key experiments. If you do not consider this to be an appropriate or feasible set of experiments to include then please detail this in your response to the reviewers' comments.

They suggest, however, that a revised version might prove acceptable, if you can address their concerns. If you think that you can deal satisfactorily with the criticisms on revision, I would be pleased to see a revised manuscript. We may then return it to the reviewers.

We are aware that you may be experiencing disruption to the normal running of your lab that makes experimental revisions challenging. If it would be helpful, we encourage you to contact us to discuss your revision in greater detail. Please send us a point-by-point response indicating where you are able to address concerns raised (either experimentally or by changes to the text) and where you will not be able to do so within the normal timeframe of a revision. We will then provide further guidance. Please also note that we are happy to extend revision timeframes as necessary.

Please ensure that you clearly highlight all changes made in the revised manuscript. Please avoid using 'Tracked changes' in Word files as these are lost in PDF conversion.

© 2022. Published by The Company of Biologists under the terms of the Creative Commons Attribution License (https://creativecommons.org/licenses/by/4.0/).

I should be grateful if you would also provide a point-by-point response detailing how you have dealt with the points raised by the reviewers in the 'Response to Reviewers' box. Please attend to all of the reviewers' comments. If you do not agree with any of their criticisms or suggestions please explain clearly why this is so.

Reviewer 1

Advance summary and potential significance to field

This Work by Roboti et al. features a thorough biochemical analysis of the interaction between the mitochondrial antiviral signalling protein and BAG6. The authors identified this physical association via BioID labelling and validated their findings using CoIP. They continue to establish the sequence of events – and more to the point: additional interacting factors – that lead to the insertion and quality control of MAVS. This work adds an important puzzle piece to our understanding of how mitochondrial TA proteins (ultimately) reach their target membrane.

Comments for the author

While I have no issue with the findings in general I do have a few points I would like the authors to consider:
(1) Figure 3D,E: I fail to understand how the difference depicted in the bar chart in 3E is established? The exemplary blot in Figure 3D is either not a good representative of the three repetitions or the result is simply a little exaggerated. The MAVS bands in the aBAG6 lanes do look far too similar (even when considered ratioed against the Bag6 bands at the bottom) for my taste to account for an almost 50% reduction. If the authors insist on this difference actually being there in vivo, I'd like to see all blots or another method to proof that SGTA is indeed needed to bridge the interaction between Bag6 and MAVS.
(2) Figure 4C: For the same reason stated under (1) – but now the other way round – I'd argue that the blot depicted in 4C shows a slightly weaker band in the aBag6 lane of siATP13A1 cell extracts and conclude that less MAVS is co-precipitated with Bag6 in absence of ATP13A1 (as opposed to equal amounts).
(3) Figure 5C: While here one can see that there is slightly more MAVS coprecipitated with Bag6 in the absence of EMC5 I wonder whether this is due to the increase in SGTA protein levels (which would support the result I am actually questioning in (1) – namely that SGTA is required (or aids) in the interaction of Bag6 and MAVS). What do the authors think about this?
(4) I have identical problems with the quantification in Figure 6B. The MAVS band at 12h post inoculation does look significantly stronger than at the zero timepoint.
Maybe I have overlooked something or misunderstand the method and their quantification. In that case I look forward to an explanation clarifying my points.

Minor points:
(5) some of the Figures would benefit from rearrangement in the order how they should be read from A to B to C etc. and not as is sometimes (Figure 3) A to D to E then continued B and C (see also Figure 5)
(6) In Figure 5i there are three blots labelled OST48. Is this correct?
(7) The cell biology in Figure 3B, C is also slightly unclear and would benefit from editing. I would also say that Figure S5 and the abundant expression of OMP25 in the pellet fraction and its almost absence from the supernatant is a stronger support for the hypothesis that the authors make on page 12 first paragraph.

Reviewer 2

Advance summary and potential significance to field

More than one third of all proteins synthesized on cytosolic ribosomes have to be transported across or inserted into subcellular membrane systems. It is truly amazing how cells manage to direct all these proteins to their destined location using a number of sophisticated (partially overlapping) targeting pathways. In the case of membrane proteins, an additional level of complexity arises from the need to shield hydrophobic domains in the aqueous environment of the

© 2022. Published by The Company of Biologists under the terms of the Creative Commons Attribution License (https://creativecommons.org/licenses/by/4.0/).

cytosol on the protein's journey. In recent years, specialized machineries and mechanisms have been described that allow for the dislocation of mistargeted proteins. However, it is unclear, if what is currently termed "mistargeting", may rather be a particularly elegant way to achieve dual targeting or preserve chances to alter the location of a protein upon an external or internal triggering stimulus.

The study presented here by Steven High's group is a very interesting contribution to this lively and competitive area of research. The authors generated an experimental system to screen for yet unknown partner proteins or substrate clients of SGTA, a co-chaperone known to bind C-terminal hydrophobic domains of membrane protein destined for the ER. Unexpectedly, amongst these clients was MAVS, a protein usually found in the mitochondrial outer membrane (MOM) that has attracted interest due its role in the innate immune response to viral infections. Further experiments show that MAVS is handed over from SGTA to the BAG6 complex, but cannot move on to the ER via the canonical downstream TRC40 route. Although the soluble BAG6-MAVS adduct in the cytosol is remarkably stable, a small fraction appears to be inserted into the ER membrane via the EMC protein insertase. If EMC is genetically ablated the BAG6-bound pool of MAVS increases. Again the question remains, if this represents a "mistargeting event" or a mechanism to potentially shuffle MAVS into mitochondria-associated ER membranes (MAM) that are thought to expand during the innate immune response in a MFN2-dependent manner. Of note, treatment of cells to mimic an innate immune response gradually reduced the amount of membrane-bound MACS in this study as reported before, whereas the newly identified soluble BAG6-bound pool remained surprisingly stable.

Although it remains possible that the BAG6-MAVS adduct represents a "dead-end intermediate" of membrane protein targeting, evidence presented in this manuscript suggests a (yet poorly understood) functional role in protein homeostasis, maybe during viral infection. This study is particularly exciting, because it raises a number of follow-up questions that are certainly beyond the scope of this first pioneering study. It will be very interesting to explore if BAG6-bound MAVS is still competent for targeting to mitochondria. The exceptional stability of this pre-targeting complex makes it tempting to speculate about a "reserve pool" of MAVS.

Comments for the author

This work is of excellent technical quality, the experiments are carefully constructed with all necessary controls and nicely build on each other. The manuscript is well written, although the authors sometimes jump forth and back between different thoughts very quickly. I have only a few minor points that I am asking the authors to address prior to publication of this overall outstanding and important work.
1) The authors discriminate only between soluble and membrane-bound MAVS throughout the manuscript, which may be understandable having in mind that they started off with a SGTA-interactor screen. However, especially in the experiments addressing the fate of MAVS in the different knock-out and knock-down cells used here, I find it appropriate to also discriminate between MOM and ER membrane localization. Mitochondrial and microsome fractions are easily separatable and should be examined separately.
2) I do not really understand the purpose of the experiment shown in figure 3B,C. My vague interpretation is that nuclear localization of TA proteins is just an artifact induced by BAG6 overexpression. Moreover, the differences are not very impressive. Is this experiment really necessary? I would at least move it to the Supplement.
3) The quality of the immunoblots in this manuscript is amazing. Naturally, this make it tempting to speculate about the potential meaning of small differences. In figure 6C, I noticed that the increase in BAG6-bound MAVS after 24 h of poly(I:C) transfection (Can you please better explain what this is?) may be accompanied by an increase in total BAG6 levels (input, lane 4). Is this reproducible and maybe meaningful?
4) Another moment of confusion I had looking at supplementary figure S7. The authors are now looking at detergent-solubilized cellular extracts leading to the transfer of virtually all MAVS to the supernatant. I assume this is done to test, if BAG6 depletion leads to the aggregation of the otherwise cytosolic MAVS pool. In any case, the purpose of this experiment should be better explained.

© 2022. Published by The Company of Biologists under the terms of the Creative Commons Attribution License (https://creativecommons.org/licenses/by/4.0/).

First revision

Author response to reviewers' comments

Point-by-point response to reviewers' comments:

We are grateful to both reviewers for their constructive comments and suggestions, and we provide a point-by-point response below. As requested, we have identified the relevant textual changes in our revised manuscript using yellow highlights.

Reviewer 1 Comments for the Author:

We thank Reviewer 1 for their conclusion that "This work adds an important puzzle piece to our understanding of how mitochondrial TA proteins (ultimately) reach their target membrane" and that they "have no issue with the findings in general".

Points 1 to 4 raised by Reviewer 1 all relate to data presented in quantitative immunoblots and we felt it would therefore be useful to summarise our general approach to these experiments before addressing each individual point. We use a LiCor system to visualise the signals emitted by secondary antibodies labelled with infrared dyes. This detection system automatically indicates whether the relevant samples are within the linear range for measurement and provides a numerical value for the intensity of the relevant signal (see Biological Repeats for Reviewers). [NOTE: The Biological Repeats for Reviewers file was provided for the referees in confidence, and has not been included in this report.]

These experiments are carried out in triplicate or more, and we appreciate that in some cases the example immunoblot provided is not identical to the graphical representation of the combined results derived from the quantification. For this reason, we have provided the Reviewers with the numerical values of the relevant immunoblot signals for all biological repeats including a full worked example for the quantification of the data presented in Figure 3 (Biological Repeats for Reviewers). We have also indicated which of these values are derived from the immunoblots that Reviewer 1 mentions in points 1 to 4 below, so that it is clear what the numerical values for the products in question are.

Points for consideration:

Point 1. Figure 3D,E: I fail to understand how the difference depicted in the bar chart in 3E is established? The exemplary blot in Figure 3D is either not a good representative of the three repetitions or the result is simply a little exaggerated. The MAVS bands in the aBAG6 lanes do look far too similar (even when considered ratioed against the Bag6 bands at the bottom) for my taste to account for an almost 50% reduction. If the authors insist on this difference actually being there in vivo, I'd like to see all blots or another method to proof that SGTA is indeed needed to bridge the interaction between Bag6 and MAVS.

Response:
We thank Reviewer 1 for raising this point and apologise for not clearly stating how these values were calculated. In the original manuscript, the amount of MAVS that was co- immunoprecipitated with Bag6 was expressed as a percentage of the total MAVS species present in the corresponding input material. In hindsight, this was not the best way to represent these data and, taking on board the comments from Reviewer 1, we have now simplified our representation of these data. In order to compare the amount of MAVS bound to Bag6 under different conditions, we now simply use the ratios of the MAVS signal (corrected for background) to the corresponding Bag6 signal (corrected for background) in the respective Bag6 IPs carried out after different treatments. By setting the control to an arbitrary value of 1.0, we believe that this comparison provides a reasonable proxy for the effectiveness of Bag6 loading after different perturbations. The graphical representation provided in Figure 3C has been updated accordingly.

We have now explained how these MAVS-Bag6 co-precipitation ratios were determined by including the following text at the end of the 'Immunoblotting' section in Material and Methods on page 22: The relative amount of MAVS in the supernatant fraction that co- immunoprecipitates with Bag6

© 2022. Published by The Company of Biologists under the terms of the Creative Commons Attribution License (https://creativecommons.org/licenses/by/4.0/).

was calculated as follows: Firstly, the quantified signals for MAVS and Bag6 species in Bag6 IPs were corrected to account for any background by subtracting the respective signals in the IgG controls. The ratio of the corrected MAVS signal to the corrected Bag6 signal was then calculated to provide a proxy for the relative amount of MAVS that was bound to Bag6 under different treatment conditions. These values were then expressed relative to the respective value obtained using control cells, which was arbitrarily set to one (see Figs 3C,4D-6D).

For the benefit of the Reviewers we provide the three original blots used to calculate the values shown in Figure 3 together with their respective quantifications. We also indicate how these values were used to generate the graph shown in Figure 3C (see Biological Repeats for Reviewers, pages 1 and 2). In each case, these quantifications suggest that the relative amount of MAVS that is recovered with Bag6 in SGTA knockout cells is reduced.

It was never our intention to overstate the role of SGTA, it is clearly dispensable given we see MAVS bound to Bag6 in SGTA knock out cells. Our working hypothesis is that SGTA may facilitate this interaction consistent with its established role in TA protein biogenesis (see pathways 2 and 3 in Figure 7). We have therefore softened our conclusions regarding the contribution of SGTA at various points in our revised text as follows:

Results section:

page 10: Section Heading now reads “MAVS exhibits a robust interaction with the BAG6 complex that may be facilitated by SGTA” *(Formerly “that is enhanced by”).*

page 11: Text now reads “Quantification of the relative amount of MAVS species that were co-immunoprecipitated with Bag6 (see Materials and Methods; Immunoblotting section) suggested a reduction in the amount of MAVS that was associated with the Bag6 protein (Fig. 3B,C).” *(Formerly “When the amounts of BAG6-bound MAVS recovered from control KO and SGTA KO cells were compared, we observed a □50% reduction following SGTA knockout (Fig. 3B,C)”).*

page 11: Text of concluding sentence now reads “that may be facilitated by SGTA” (*Formerly “that can be facilitated by SGTA*).

Discussion section:

page 15: Text now reads “In further support of this model, we recover less Bag6-bound MAVS in SGTA KO cells as compared to control cells (Fig. 3B,C).” *(Formerly “we find that the proportion of MAVS recovered with the BAG6 complex is reduced upon SGTA knockout (Fig. 3).)”.*

page 15: Text now reads “Nevertheless, SGTA is clearly dispensable for MAVS binding to the BAG6 complex” *(Formerly “Nevertheless, SGTA is dispensable for MAVS binding to the BAG6 complex”).*

Point 2. Figure 4C: For the same reason stated under (1) – but now the other way round – I’d argue that the blot depicted in 4C shows a slightly weaker band in the aBag6 lane of siATP13A1 cell extracts and conclude that less MAVS is co-precipitated with Bag6 in absence of ATP13A1 (as opposed to equal amounts).

Response:
We now provide the quantification data obtained from all four biological replicates (see Biological Repeats for Reviewers, pages 3 and 4) and these data confirm that across the four separate experiments carried out we found no evidence that knockdown of ATP13A1 had a significant effect on the amount of MAVS recovered with Bag6 via co-immunoprecipitation. We do however acknowledge that the original immunoblot that we chose to illustrate the cumulative data shown in the Figure 4D was not the best choice we could have made. We have therefore replaced Figure 4C with a more representative set of immunoblots (Biological Repeats for Reviewers, page 4, Experiment 3). Likewise, the graphical representation of these data shown in Figure 4D has been updated as described for Figure 3C in our response to Reviewer 1, Point 1 above.

Point 3. Figure 5C: While here one can see that there is slightly more MAVS coprecipitated with Bag6 in the absence of EMC5 I wonder whether this is due to the increase in SGTA protein levels

© 2022. Published by The Company of Biologists under the terms of the Creative Commons Attribution License (https://creativecommons.org/licenses/by/4.0/).

(which would support the result I am actually questioning in (1) – namely that SGTA is required (or aids) in the interaction of Bag6 and MAVS). What do the authors think about this?
Response:
We have quantified the relative SGTA levels in the input samples for the four repeats carried out to provide the data shown in Figure 5D and we find no evidence for a difference in SGTA levels following EMC5 knockdown (see bar graph).

[NOTE: We have removed a figure which was provided for the referees in confidence.]

As for Figures 3C and 4D, the graph for Figure 5D has been updated. As discussed above, on the basis of the differences that we see between control and SGTA knockout cells (Figure 3), our conclusion is that SGTA may facilitate the binding of MAVS to Bag6, but it is NOT essential for this process (see also response to point 1).

Point 4. I have identical problems with the quantification in Figure 6B. The MAVS band at 12h post inoculation does look significantly stronger than at the zero timepoint. Maybe I have overlooked something or misunderstand the method and their quantification. In that case I look forward to an explanation clarifying my points.

Response:
Firstly, we apologise for not clearly stating how the percentage of membrane-associated (Fig. 6B) and cytosolic MAVS (Figs 4B-6B) were calculated. We have now included the following text on page 22 of our revised manuscript: Quantification of cytosolic and membrane- associated MAVS was performed as follows: MAVS signals in the supernatant and pellet fractions were normalised to tubulin and OST48 loading controls, respectively. The ratio of the normalised MAVS signal in each fraction to the total MAVS signal was then calculated, and the resulting value was expressed relative to the respective value in control cells (see Figs 4B-6B). Furthermore, we now include the individual data points for the bar graphs shown in Figure 6, something we had omitted to do in our original manuscript. This revised Figure shows a fairly large spread for these values (Figure 6Bii) particularly at the later time points. Statistical analysis of the data indicates that there is no significant difference in the pool of cytosolic MAVS across these different time points.

For the benefit of the Reviewers, we also provide blots from all our six biological replicates together with their respective quantifications and show how these values were used to generate the graphs shown in Figure 6B (see Biological Repeats for Reviewers, pages 7-9).

Minor points:

Point 5. Some of the Figures would benefit from rearrangement in the order how they should be read from A to B to C etc. and not as is sometimes (Figure 3) A to D to E then continued B and C (see also Figure 5).

Response:
Based on point 7 from Reviewer 1 and point 2 from Reviewer 2, we have now removed Figures 3B,C of the original manuscript and the labelling of the panels shown in the revised version of Figure 3 is now consecutive. In the case of Figure 5, we have rearranged the panels as suggested.

Point 6. In Figure 5i there are three blots labelled OST48. Is this correct?

Response:
We thank Reviewer 1 for flagging the lack of clarity regarding OST48 and apologise for this oversight. The blots are correct as displayed, with OST48 acting as a loading control for membrane fractions that were resolved on separate gels prior to analysis. We have now added the following text to the legend accompanying Figure 5B on page 39 of our revised manuscript: "Blots resulting from the same membrane are clustered together. Tubulin and OST48 serve as loading controls for the supernatant and pellet fractions, respectively."

Point 7. The cell biology in Figure 3B,C is also slightly unclear and would benefit from editing. I would also say that Figure S5 and the abundant expression of OMP25 in the pellet fraction and its almost absence from the supernatant is a stronger support for the hypothesis that the authors make

© 2022. Published by The Company of Biologists under the terms of the Creative Commons Attribution License (https://creativecommons.org/licenses/by/4.0/).

on page 12 first paragraph

Response:
Given the comments of Reviewer 1, and following the direct suggestion of Reviewer 2 (see their point 2), we have now deleted panels B and C of Figure 3 and removed the accompanying text in the Figure legend and results section of our updated manuscript. Our results section now focuses on the properties of OMP25 and MAVS in parental (Fig. S4B) and control KO (Fig. S5A) HepG2 cells as suggested by Reviewer 1. Our original text already highlighted that OMP25 was recovered almost exclusively in the pellet fraction, and we have kept this text in our revised manuscript whilst now including a reference to the data shown in Figure S5, see page 10: "consistent with our fractionation studies which suggest that the majority of OMP25 is membrane inserted at steady state (Figs S4B,S5A)".

Reviewer 2 Comments for the Author:

We thank Reviewer 2 for their positive comments and their assessment of our manuscript as "overall outstanding and important work". They concluded that they "have only a few minor points that I am asking the authors to address prior to publication".

Point 1. The authors discriminate only between soluble and membrane-bound MAVS throughout the manuscript, which may be understandable having in mind that they started off with a SGTA-interactor screen. However, especially in the experiments addressing the fate of MAVS in the different knock-out and knock-down cells used here, I find it appropriate to also discriminate between MOM and ER membrane localization. Mitochondrial and microsome fractions are easily separatable and should be examined separately.

Response:
We agree that the distribution of MAVS between different membrane fractions is an important consideration, but we respectfully suggest that these experiments are beyond the scope of our current manuscript. The focus of our study here was to try to characterise the nature of a previously unidentified soluble pool of MAVS that is bound to BAG6 and we believe that we provide credible evidence for our current working hypothesis, as presented in Figure 7. It is also worth mentioning that MAVS is also found at the peroxisomal membrane, in addition to the MOM and ER (Dixit et al., 2010). In short, there is no doubt that such experiments have merit, but we believe that such work is something that can be better addressed in future studies.

Point 2. I do not really understand the purpose of the experiment shown in figure 3B,C. My vague interpretation is that nuclear localization of TA proteins is just an artifact induced by BAG6 overexpression. Moreover, the differences are not very impressive. Is this experiment really necessary? I would at least move it to the Supplement.

Response:
Whilst we have used this non-physiological "re-localisation" approach in previous studies of tail-anchored proteins, neither Reviewer 1 nor Reviewer 2 found that the results presented in Figure 3B,C added anything substantial to our biochemical studies of the interactions between BAG6 and MAVS, Stx-5 and OMP25. We have therefore completely removed these data and the accompanying text completely from our revised manuscript

Point 3. The quality of the immunoblots in this manuscript is amazing. Naturally, this makes it tempting to speculate about the potential meaning of small differences. In Figure 6C, I noticed that the increase in BAG6-bound MAVS after 24 h of poly(I:C) transfection (Can you please better explain what this is?) may be accompanied by an increase in total BAG6 levels (input, lane 4). Is this reproducible and maybe meaningful?

Response:
Polyinosinic-polycytidylic acid (poly(I:C)) is a synthetic analogue of viral dsRNA and its delivery into the cytoplasm by transfection is frequently used to activate the MAVS-dependent immune response pathway. We now spell this out at the first mention of this technique by modifying the text on page 13 so that it now reads ''To test this, we transfected control or Bag6-depleted cells with high-

© 2022. Published by The Company of Biologists under the terms of the Creative Commons Attribution License (https://creativecommons.org/licenses/by/4.0/).

molecular weight polyinosinic-polycytidylic acid (poly(I:C)), which mimics the intracellular dsRNA generated during viral replication and specifically activates the MDA5 RIG- I-like receptor (Kato et al., 2008), and examined…..''

Our analysis of multiple experiments reveals a transient decrease in BAG6-bound MAVS at 4 and 12 hours after poly(I:C) transfection, but by 24 hours post-treatment the levels of BAG6- bound MAVS have returned to their initial level as Reviewer 2 suggests (Fig. 6C,D; see also quantification data obtained from all six biological replicates in Biological Repeats for Reviewers, pages 10-12). In order to address Reviewer's 2 question about total Bag6 levels, we have quantified these for all time points in all six biological repeats and provide the results in the bar graph below, shown relative to time zero which is set to an arbitrary value of 1.0.

[NOTE: We have removed a figure which was provided for the referees in confidence.]

Although there is a greater spread of values at the later time points, the results do not support the suggestion that there is a significant increase in Bag6 levels after 24 hours.

It is this finding that leads us to conclude that "These data show that the population of MAVS that is bound to the BAG6 complex is dynamic and may respond to the activation of innate immune signalling following viral infection" at the end of our Discussion (see page 18). We do indeed believe that these data are relevant to the physiological role of MAVS and hope that future studies will address the role of the soluble pool of MAVS that we reveal in this study.

Point 4. Another moment of confusion I had looking at Supplementary Figure S7. The authors are now looking at detergent-solubilized cellular extracts leading to the transfer of virtually all MAVS to the supernatant. I assume this is done to test, if BAG6 depletion leads to the aggregation of the otherwise cytosolic MAVS pool. In any case, the purpose of this experiment should be better explained.

Response:
Our apologies for oversimplifying here. Reviewer 2 is correct that we were actually looking at both total protein levels and the solubility of MAVS here. Since we saw no evidence for increased aggregation, we did not mention this in our original text. We have now rectified this omission in our revised version by expanding the relevant text on page 16 to state that "Knockdown of the Bag6 protein has no clear effect on the steady-state levels of MAVS, nor does it result in the formation of detergent-insoluble MAVS aggregates (Fig. S7). We have also changed the title of Fig. S7 to "Bag6 deficiency does not affect steady-state MAVS levels or solubility".

Second decision letter

MS ID#: JOCES/2021/259596

MS TITLE: Mitochondrial antiviral-signalling protein is a client of the BAG6 protein quality control complex

AUTHORS: Peristera Roboti, Craig Lawless, and Stephen High
ARTICLE TYPE: Research Article

I am happy to tell you that your manuscript has been accepted for publication in Journal of Cell Science, pending standard ethics checks. Given the positive comments from the reviewers' initial evaluation and the careful and well explained revisions you have made, I did not consider it necessary to return this to the reviewers again. Congratulations to you and your co-authors on a nice piece of work.

© 2022. Published by The Company of Biologists under the terms of the Creative Commons Attribution License (https://creativecommons.org/licenses/by/4.0/).
